# Surface warming reacceleration in offshore China and its interdecadal effects on the East Asia–Pacific climate

**DOI:** 10.1038/s41598-020-71862-6

**Published:** 2020-09-09

**Authors:** Yulian Tang, Jingliang Huangfu, Ronghui Huang, Wen Chen

**Affiliations:** 1 Center for Monsoon System Research, Institute of Atmospheric Physics, Chinese Academy of Sciences, Beijing, 100190 China; 2 University of Chinese Academy of Sciences, Beijing, 100049 China

**Keywords:** Atmospheric dynamics, Physical oceanography

## Abstract

Since the late 1970s, sea surface temperatures (SSTs) have exhibited greater responses to global warming in the offshore area of China and adjacent seas (offshore China) than in the global ocean. This study identified a surface warming reacceleration in offshore China since 2011, following a well-known interdecadal shift from offshore surface warming to cooling in 1998. During the warming reacceleration period, the rate of increase in offshore China SSTs was twice the mean rate of global ocean surface warming, and the significantly warming area was primarily in the north, especially in the East China Sea. Concurrent with the ascending phase of the Interdecadal Pacific Oscillation, a large area of positive sea level pressure anomalies developed over the tropical Pacific. Accordingly, the surface southerly wind anomalies contributed to the recent surface warming in offshore China, especially in the East China Sea. With greater changes in the warming rate, the spatial mode of the circulation anomalies over East Asia and the western Pacific has shifted westward and has exerted more inshore influence during the recent warming reacceleration period than during the previous periods.

## Introduction

Global warming is explicitly based on the fact that the global mean temperatures of both land and ocean surfaces have increased by 0.85 °C from 1,880 to 2012^1^. However, global warming has not proceeded monotonously with the steady increase in greenhouse gas emissions but has instead exhibited two pronounced patterns: warming acceleration and warming slowdown^2,3^.

The period from the mid-to-late 1970s to the late 1990s featured the greatest global warming acceleration since 1900^2,3^. Many resultant unprecedented phenomena, including declining ice cover, melting glaciers, rising sea levels, the extinction of endemic species, and the aggravation of eutrophication, have been reported worldwide^4–8^. The ocean buffers surface warming by absorbing both heat and carbon dioxide, which increases most sea surface temperatures (SSTs)^9^.

The slowdown in the warming of the global mean surface air temperature (GMST) since 1998 has attracted great attention^3,10^, and slowing has also been observed in global mean SST warming^11–13^. Dong and Dai^14^ indicated that a cooling trend over the equatorial Central and Eastern Pacific has been observed since the late 1990s and that this trend is associated with the switch in the Interdecadal Pacific Oscillation (IPO) or its North Pacific counterpart (the Pacific Decadal Oscillation) from a warm phase to a cold phase in approximately 1999. Liao et al.^15^ revealed that the mean warming trend of global coastal SSTs has been lower during the warming slowdown period than during the warming acceleration period and that some coastal warming trends have even reversed in the Pacific Ocean, North Indian Ocean, part of the North Atlantic and most of the South Atlantic since 1998.

Recently, the years 2014, 2015 and 2016 have consecutively broken the highest temperature record, and the past 5 years have become the warmest years on record since 1880^16^. There is a growing consensus that the warming slowdown ended after 2010^3,17–21^. Recently, Zhang et al.^20^ claimed that the global warming slowdown dissolved in the early 2010s and explained that the combined contributions of the decadal-to-multidecadal component and the long-term warming trend show greater contributions than the interannual component, implying that the warmer years observed recently may occur more frequently in the near future. They suggested that global warming has entered a new stage based on the positive phase of the Pacific Decadal Oscillation (PDO) and Atlantic Multidecadal Oscillation (AMO) and the increasing trend in the North Atlantic Oscillation index.

There is a regionality to the ocean surface warming rate due to discrepancies in atmospheric circulation and ocean currents and the heterogeneous distribution of aerosols^22–24^. Previous studies have investigated the regional climate, marine environment and ecosystem in the offshore areas of China and adjacent seas (hereafter, offshore China) during the two periods of global warming^25–30^ because this region is an important component of coastal boundary systems, which provide 10.6% of primary production and 28.0% of global fishery production^24^. Since the late 1970s, the SST in offshore China (including the East China Sea (ECS) and South China Sea (SCS)) has rapidly increased^25–27,29,30^, and the warming rate has been approximately five times as fast as the global mean SST warming rate during the warming acceleration period^28^. In particular, the broad ECS (23°N–40°N, 120°E–130°E; including the Bohai Sea, Yellow Sea, and East China Sea) shows more robust warming with extreme SST events^30^. Furthermore, a substantial increase in the SST can induce relatively increased (decreased) proportions of warm-water (warm-temperate) zooplankton and fish species and the easier occurrence of phytoplankton blooms and harmful algae blooms, which increase the vulnerability of the offshore ecosystem and pose risks to local fisheries^26,29^. However, an inverse cooling trend appeared in offshore China during the global warming slowdown period^28–30^.

Since offshore China is a region that is highly sensitive to global climate change and exerts a vital influence on the local marine ecosystem and climate, it is essential to study the response of the offshore China SST to the recent global warming reacceleration and its impacts in the context of global warming. Consequently, a focused investigation of the climatic effects of global warming reacceleration on offshore China is required.

## Results

### Different warming trends during three periods

**Figure 1:**
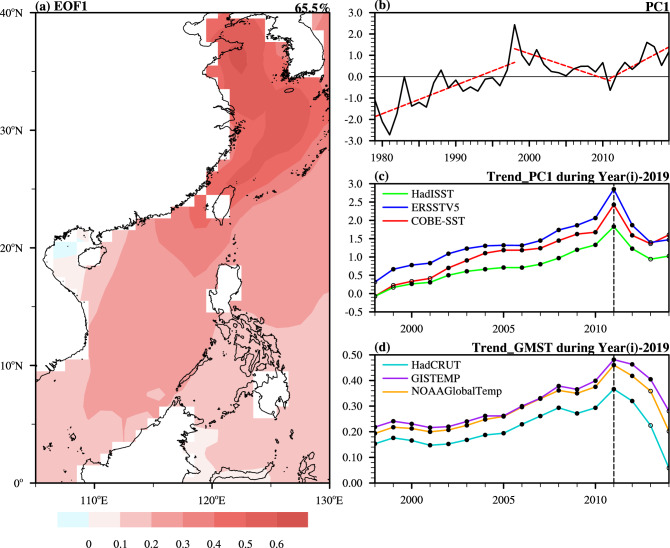
(**a**) The leading EOF1 for the annual mean SSTAs in offshore China during 1979–2019 and (**b**) its PC1 based on the HadISST dataset. (**c**) The linear tendencies of PC1 from the HadISST, ERSSTV5 and COBE-SST datasets with different time interval selections. (**d**) The linear tendencies of the annual GMST anomalies from the HadCRUT, GISTEMP and NOAAGlobalTemp datasets with different time interval selections. Each year on the X-axis in (**c**) and (**d**) denotes the period between that year and 2019 for the linear trend analysis. The solid (hollow) circles indicate that the trend is (is not) significant at the 90% confidence level according to a two-sided Student's *t* test. The map in figure (**a**) is generated using the NCAR Command Language (Version 6.6.2) [Software]. (2019). Boulder, Colorado: UCAR/NCAR/CISL/TDD. 10.5065/D6WD3XH5.

Figure 1a,b present the spatial–temporal variable characteristics of the SST in offshore China based on the 1979–2019 data from the Hadley Centre Sea Ice and Sea Surface Temperature (HadISST^31^) dataset. Accounting for 65.5% of the total variation, the leading empirical orthogonal function (EOF1) mode (Fig. 1a) reflects the primary variability in the SST anomaly (SSTA). The EOF1 SSTA exhibits a spatial in-phase variation, which is stronger at high latitudes than at low latitudes, larger inshore than offshore, and most significant in the ECS. Its principal component (PC1) (Fig. 1b) shows robust interannual and interdecadal variations, with a warming trend from the late 1970s to the late 1990s and a cooling trend after approximately 1998, which is consistent with the findings of Tan et al.^28^ and Cai et al.^30^. Moreover, as is clear from Fig. 1b, there was a warming regime shift in approximately the early 2010s. To detect the recent interdecadal reacceleration in the offshore China SST more objectively, we utilized two more SST datasets, including the National Oceanic and Atmospheric Administration (NOAA) Extended Reconstructed Sea Surface Temperature V5 (ERSSTv5^32^) dataset and the Centennial Observation-Based Estimates of the Variability of Sea Surface Temperature (COBE-SST^33^), and employed the method proposed by Yao et al.^2^, which has been applied successfully to distinguish distinct global warming periods. Considering that 1998 is usually regarded as the starting year of the warming slowdown, we set the first detection year as 1998 (X-axis in Fig. 1c), and each year on the X-axis represents the period between that year and 2019. Each value on the Y-axis shows the linear trend of the corresponding period, and the solid (hollow) circle denotes that the trend is (is not) significant at the 90% confidence level. As shown in Fig. 1c, the maximum significant tendency was observed in 2011 based on the three SST datasets, meaning that a significant interdecadal warming trend can be identified since 2011. In addition, the global warming rate has experienced similar interdecadal fluctuation since the late 1970s, as characterized by an acceleration from the late 1970s to the late 1990s, a slowdown from the late 1990s to the early 2010s, and a reacceleration in recent years^3^. As an inspiration for the present study, we are also curious about whether the SST in offshore China changed synchronously with the GMST during the recent global warming reacceleration. We performed a similar linear trend analysis on the annual GMST anomalies (Fig. 1d) based on the three most highly cited merged land–ocean temperature datasets: the Hadley Centre Climate Research Unit Temperature Dataset (HadCRUT^34^), the Goddard Institute for Space Studies Surface Temperature Dataset (GISTEMP^35^) and the NOAA Global Surface Temperature Dataset (NOAAGlobalTemp^36^). In Fig. 1d, a significant maximum warming tendency was observed during 2011–2019 among all three datasets, suggesting that the global warming reacceleration based on the GMST started in 2011. Hence, both the offshore China SST and the GMST shared the turning year (2011) and entered the rewarming period synchronously. Similar interdecadal variation also occurred in the seasonal offshore China SSTs (Supplementary Figs. 1–4). The results show that the boreal wintertime variance contributed a larger percentage to the total variance in EOF1, as has been revealed previously^30,37–40^. In addition, although all four seasons show large transitions in approximately 2011, the warming trends are more significant during boreal spring, summer and winter than during autumn. The significance of the signals is expected to increase when specific studies are conducted during these seasons.

**Figure 2:**
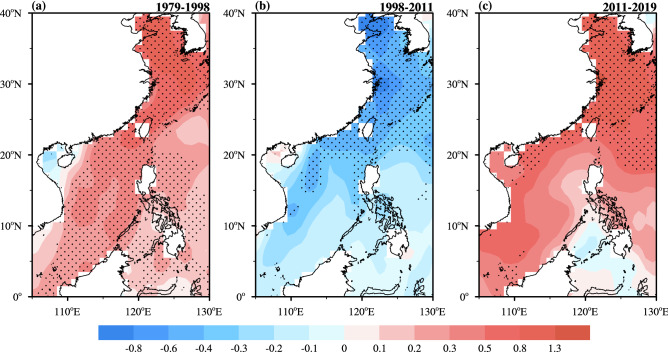
Spatial patterns of the linear trends (unit: °C/decade) of the annual mean SSTAs in offshore China during (**a**) the warming acceleration period (1979–1998), (**b**) the warming slowdown period (1998–2011) and (**c**) the warming reacceleration period (2011–2019). Stippling denotes 10% significance according to a two-sided Student's *t* test. The maps in the figure are generated using the NCAR Command Language (Version 6.6.2) [Software]. (2019). Boulder, Colorado: UCAR/NCAR/CISL/TDD. 10.5065/D6WD3XH5.

In the following sections, this study adopts the terms warming acceleration period^2,15^ and warming slowdown period^2,11,38^ to denote the epochs of 1979–1998 and 1998–2011, respectively, and refers to the epoch of 2011–2019 as the warming reacceleration period. Figure 2 shows the magnitude distribution of the linear SST changes in offshore China and displays notable contrasts among the three periods. During the warming acceleration period, most offshore China waters have a significant warming trend (~ 0.31 °C/decade), and the fastest-warming region is located in the northwestern region of the ECS, with a rate of 0.80–1.30 °C/decade, far exceeding the mean rate of global ocean surface warming (~ 0.07 °C/decade) during the same period. In contrast, the offshore China SST decreases at a rate of − 0.27 °C/decade during the warming slowdown period, while the global mean SST appears to stall (~ 0.03 °C/decade). The relative relation is similar to the relationship discovered by Tan et al.^28^—the surface warming rate in offshore China is approximately five times greater than the global mean SST warming rate during the warming acceleration period, while the apparent cooling in offshore China is in sharp contrast with the leveling off of the global mean SST during the warming slowdown period. However, the absolute surface warming rates in offshore China during these two periods are less than those of Tan et al.^28^ due to the different SST datasets used.

Compared to the previous two periods, the linear SST variation in offshore China displays a similar spatial pattern but a larger magnitude (~ 0.45 °C/decade), which is almost twice the rate of global ocean surface warming (~ 0.24 °C/decade) (Fig. 2c). We also checked the warming reacceleration period based on different seasons (Supplementary Fig. 5). The distribution of the linear trends of the seasonal offshore China SSTAs shows strong resemblances to that in Fig. 2c, with robust warming trends in offshore China, especially in the ECS. To confirm our analysis displayed in Figs. 1 and 2 indicating that surface warming has intensified in offshore China since 2011, our study further examines other SST datasets, including the ERSSTv5 and COBE-SST datasets. Consistent results among the three SST datasets confirmed this warming reacceleration since 2011 (Supplementary Figs. 6–9).

**Figure 3:**
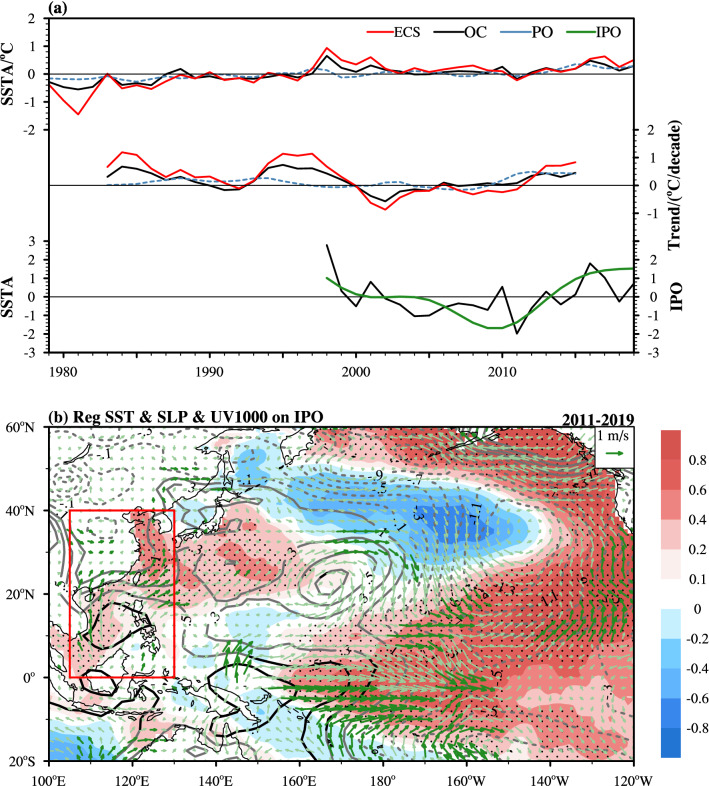
(**a**) Time series of the annual mean SSTAs (upper, unit: °C) and their 9-year moving linear trends (middle, unit: °C/decade) in the ECS (23°–40°N, 120°–130°E; red solid line), offshore China (OC; 0°–40°N, 105°–130°E; black solid line) and the Pacific Ocean (PO; 40°S–60°N, 105°E–70°W; blue dashed line) during 1979–2019, and normalized time series (lower, unit: 1) of the IPO index (green solid line) and the annual mean SSTA in offshore China (black solid line) during 1998–2019. (b) Spatial distributions of the regression of the annual SST (shaded; unit: °C), SLP (line; unit: hPa) and 1,000-hPa field (vector; unit: m/s) on the IPO index during the warming reacceleration period (2011–2019). The red box indicates the coverage of offshore China (0°–40°N, 105°E–130°E). The black dots, lines and dark green vectors denote 10% significance according to a two-sided Student's *t* test. The map in figure (**b**) is generated using the NCAR Command Language (Version 6.6.2) [Software]. (2019). Boulder, Colorado: UCAR/NCAR/CISL/TDD. 10.5065/D6WD3XH5.

Note that the SSTAs in the north are more sensitive to climate change than those in the south of offshore China. The most prominent warming trends are also observed in the north (Fig. 2a,b), not only in this recent warming reacceleration period (Fig. 2c). The warmer SSTs (above 28 °C) are located to the south of 20°N and SSTs decrease rapidly beyond 20°N. Hence, the ECS region (23°N–40°N, 120°E–130°E) features a large temperature gradient and high standard deviation (figure not shown). This signal-amplifying effect is consistent with the findings of previous studies (e.g., Cai et al.^27,29^; Tan et al.^28^). To explain the evolution of the SST trend in offshore China, especially in the ECS, the time series of SSTAs and their 9-year moving linear trends during 1979–2019 are delineated in Fig. 3a. In offshore China, the SST increased rapidly until 1998, with the maximum warming rate in the mid-1980s and mid-1990s. From 1998 to 2011, the SST trend is negative overall, with the maximum cooling rate in 2002, and then it switches to positive again. The SSTs of the ECS and Pacific Ocean (40°S–60°N, 105°E–70°W) exhibit synchronous interdecadal fluctuations with magnitudes and rates that are larger and smaller, respectively, than those of the offshore China SST during these three time periods. In other words, the SST in offshore China, especially that in the ECS, is more sensitive to global warming than that in the Pacific Ocean. For example, during the warming reacceleration period, the linear tendencies of the SSTs in the ECS, offshore China and the Pacific Ocean are 0.83 °C/decade, 0.45 °C/decade and 0.42 °C/decade, respectively.

### The underlying mechanisms of the warming trends in the reacceleration period

The area-averaged SST time series for offshore China and the Pacific Ocean (Fig. 3a) are significantly correlated, with a correlation coefficient of 0.68 during 1979–2019, implying that the SST anomalies in offshore China change synchronously with those in the Pacific basin, which is consistent with the results of previous studies^37,40^. Considering that the IPO is the dominant form of interdecadal variability in the Pacific Ocean, we investigated the correlation between the IPO and the 9-year low-pass-filtered SST variation in offshore China. During the recent two warming stages (1998–2019), the correlation was found to be significant with a coefficient of 0.85. As shown in Fig. 3a, the normalized IPO index and annual offshore China SSTA shared a similar downward linear trend during the warming slowdown period (1998–2011) and a similar upward linear trend during the warming reacceleration period (2011–2019).

As shown in Fig. 3b, we regressed the annual sea level pressure (SLP), 1,000-hPa wind field and SST onto the IPO index during the warming reacceleration period. The SST anomalies regressed on the ascending IPO phase are characterized by positive SST anomalies in the central and eastern tropical Pacific and the western coast of North America and negative SST anomalies in the middle of the western North Pacific and central North Pacific. Concurrently with the ascending phase of the IPO (Fig. 3a), cool SSTs to the east of the Philippines induced a large area of positive SLP anomalies in the tropical Pacific. As these anomalies are of greater interest, our present study focused on the enhanced SLP over the SCS and the Philippines (red box). Additionally, the surface wind field shows a significant signal around the western and eastern rims of the abnormally high-pressure region (7°N–18°N, 108°E–126°E). The significant northward wind anomalies (dark green vectors) reach 30°N, with less significant northward winds to the north. As shown in Supplementary Figs. 10a and 10b, significant wind anomalies can lead to an enhancement in the Kuroshio Current, especially in the region along the ECS shelf, based on ocean current data from the Simple Ocean Data Assimilation (SODA 3.4.2) system (Carton et al.^41^). Additionally, we examined the velocity of the original winds during the warming reacceleration period and found significantly weaker wind velocity anomalies in the 18°N–35°N, 123°E–130°E region (Supplementary Fig. 10c), which also contributed to surface warming in the northern part of offshore China through the wind-evaporation-SST effect. Therefore, both processes have contributed to warming in northern offshore China. Considering the high interannual correlation between the offshore China SSTA and the IPO index and the above regression analysis, we consider the IPO to be the dominant cause of the recent warming reacceleration. In addition, the warming reacceleration trend of the offshore China SSTA (Figs. 1b and 2c) can be attributed to the developing trend of the IPO warm phase (Fig. 3a). Concurrent with the warming trend in the Pacific basin, the offshore China SST has exhibited a consistent long-term warming trend during 2011–2019.

As revealed by previous studies^37,39,40,42–44^, SST changes in other basins can also exert important influences on regional ocean warming. In Table 1, we show the correlation coefficients between the offshore China SSTA and the important indices over the Pacific (IPO, PDO, North Pacific Oscillation (NPO), and El Niño-Southern Oscillation (ENSO)), the Atlantic (AMO), the Arctic Ocean (Arctic Oscillation (AO)) and the Indian Ocean (Indian Ocean Dipole (IOD)) during 2011–2019. The results showed that the three oscillations with long-term periods were highly connected with the recent warming reacceleration, including the IPO (0.77), AMO (0.73) and PDO (0.62). Previous studies (e.g., Deser et al.^45^; Dai et al.^46^; Meehl et al.^47^) treated the PDO as the northern component of the IPO, considering that the selected domain of the PDO belongs to that of the IPO. The warming reacceleration in offshore China involves warming SSTs from the tropics; hence, a more integrated (including the tropical Pacific Ocean) oscillation (IPO) shows a higher correlation coefficient than the PDO. In addition, the AMO may be associated with warming reacceleration in offshore China through a teleconnection mechanism. Sun et al.^48^ summarized the remote forcing mechanisms of the AMO on the SST over the Pacific (including offshore China) on a multidecadal timescale. AMO-related warm SSTs can generate wind anomalies and decrease the westerlies over the subtropical North Pacific, leading to long-term SST warming through the wind-evaporation-SST effect. Furthermore, the warming extended southward following the SST-longwave radiation feedback mechanism. In contrast, the interannual factors showed insignificant correlations, and ENSO (0.43), AO (− 0.34) and IOD (− 0.25) events may show modulation in specific years (such as strong El Niño events during 2014–2016), while the NPO showed a weak signal. In addition, according to some recent research^20,49^ and our analysis (Fig. 1c), the global warming hiatus has faded away, and the past 10 years are the top ten warmest years on record in terms of the historical ocean heat content recorded with modern instruments. Therefore, the present study prefers to treat the recent warming trend as a response to global warming, and the recent warming reacceleration response can be attributed to both the IPO and the joint effects from the other ocean basins.

**Table 1:** Correlation coefficients between climate indices and the annual mean SSTA in offshore China.

Epoch	IPO	AMO	PDO	ENSO	AO	IOD	NPO
2011–2019	0.77**	0.73**	0.62*	0.43	− 0.34	− 0.25	− 0.03

**(*) represents the 95% (90%) confidence level according to a two-sided Student's *t* test.

### The effects of interdecadal SST variation on the East Asia–Pacific climate

**Figure 4:**
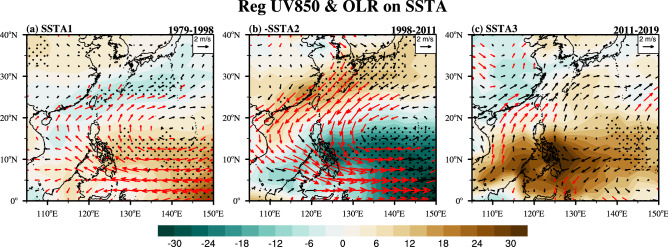
Spatial distribution of the regression of the 850-hPa wind field (vector; unit: m/s) and OLR field (shaded; unit: W/m^2^) on the annual mean SSTA in offshore China during (**a**) the warming acceleration period and (**c**) the warming reacceleration period. (**b**) is the same as (**a**) and (**c**) but for − 1*SSTA. The dots and red vectors denote regression significance at the 90% confidence level. The maps in the figure are generated using the NCAR Command Language (Version 6.6.2) [Software]. (2019). Boulder, Colorado: UCAR/NCAR/CISL/TDD. 10.5065/D6WD3XH5.

The surface thermal state of the regional ocean dominates the supply of heat and moisture in the atmosphere. Hence, the variation in the local SST may exert a significant influence on the regional atmospheric circulation and convective activities^50,51^. Figure 4 depicts the regression maps of the 850-hPa horizontal wind fields and the outgoing longwave radiation (OLR) fields, which are regressed onto the SSTA time series during the aforementioned three warming periods over offshore China. During the warming acceleration period (Fig. 4a), the enhancements in the tropical easterlies and western Pacific subtropical high (WPSH) are significant at a 90% confidence level, in contrast with the weakening of the tropical easterlies and the WPSH during the warming slowdown period (Fig. 4b). The 9-year moving linear trends of the offshore China SSTA are negative during the warming slowdown period (Fig. 3a), which indicates persistent cooling on the interdecadal timescale. Therefore, we multiplied the data by − 1 in Fig. 4b. The OLR and low-level wind anomalies reflect changes occurring under the SST anomalies with a cooling trend. Similarly, the response of the OLR between the two periods is characterized by a predominant southeast- and northwest-oriented tripole pattern with positive or negative convection anomalies over the northern SCS and southern ECS and adjacent continent to the south of the Yangtze estuary (Fig. 4a,b).

Different from the case in the previous two periods, distinct meridional wind anomalies at 850 hPa are observed during the warming reacceleration period (Fig. 4c), when the significant warming center shifts to the north (Fig. 2c). The southerly wind component is enhanced over the SCS and to the north of the Philippines, and anomalous cyclonic circulation is observed over mainland China. In addition, the spatial distribution of the regressed convection shows a dipole pattern, characterized by enhanced (depressed) anomalies located over the continent (sea). The wind anomalies favor warm-moist flows from the Bay of Bengal and the SCS, which are beneficial to the genesis and development of precipitation in East China. The differences between Fig. 4b,c show the interdecadal changes in the spatial modes of the circulation patterns over East Asia and the western Pacific. The location of the subtropical high over the western Pacific shifts significantly westward in Fig. 4c. Accordingly, significant wind anomalies are observed over East China, bringing more inshore influence than that during the previous period. In addition, the influences on South Korea, Japan and the Philippines are also remarkably different. Note that the spatial structures in Fig. 4c show a strong resemblance to the regressions onto the IPO time series (Supplementary Fig. 11). However, more regional details cannot be found in the IPO regression results, such as the significantly enhanced northward wind anomalies over the SCS and to the north of the Philippines. With greater warming change rates, SST anomalies in offshore China have shown regional modulation of the influence of the IPO. The local wind and OLR anomalies in offshore China showed greater significance to the south of the ECS, where there is a belt of transition between warm and cold SSTs. According to previous studies (e.g., Gray^52^), when the SST reaches 26.5 °C, significant convection can be induced. Therefore, the OLR and wind anomalies in this region are more sensitive to changes in SSTs. In addition, the timescale length of the data employed in our study may also influence the significance.

## Discussion

This study examined the interdecadal variation in the offshore China SST and its impact on the East Asia–Pacific climate during 1979–2019 under global warming. The interdecadal changes in the offshore China SST were characterized by surface warming during the warming acceleration period (1979–1998), followed by surface cooling during the warming slowdown period (1998–2011) and strong surface warming during 2011–2019 (referred to as the warming reacceleration period). This interdecadal variation was generally in sync with the variation in the GMST. In addition, the offshore China SST (from 0.31 °C/decade to − 0.27 °C/decade and then to 0.45 °C/decade), particularly the ECS SST, showed stronger responses to global warming than the global mean SST (from 0.07 °C/decade to almost zero and then to 0.24 °C/decade) during the above three periods.

The recent warming reacceleration trend in offshore China can be largely attributed to the developing IPO. First, the offshore China SST switched from a cooling trend to a warming trend in 2011, almost synchronously with the switch in the IPO from the descending phase to the ascending phase (Fig. 3a). Second, the 9-year moving linear trends of the SST in offshore China were basically equivalent to those in the Pacific Ocean (Fig. 3a). Third, the warming process can be explained by IPO-induced surface southerly wind anomalies through wind-driven ocean currents and the wind-evaporation-SST effect. Specifically, cool SSTs to the east of the Philippines created a large area of positive SLP anomalies over the tropical Pacific in the ascending phase of the IPO. The enhanced SLP over the SCS and the Philippines led to surface southerly wind anomalies and, thus, the northward intrusion of warmer SSTs (Fig. 3b; Supplementary Figs. 10a and 10b). In addition, the southerly wind anomalies are opposite to the climatological northerly winds, subsequently weakening the wind speeds in the 18°N–35°N, 123°E–130°E region. The weakened wind also contributed to the warming in the northern part of offshore China through the wind-evaporation-SST effect (Supplementary Fig. 10c). Therefore, concurrent with the ascending phase of the IPO, the interannual highly correlated offshore China SST exhibits a consistent warming trend with the Pacific SST during 2011–2019.

As revealed by previous studies^53–55^, although the Pacific SSTs play essential roles in global warming, SST changes in other basins can also exert important influences on regional warming rates in specific years. For example, a negative IOD can lead to anticyclonic circulation anomalies over the western North Pacific, promoting enhanced downward solar radiation and the northward intrusion of the Kuroshio Current, thus prompting warming events in the ECS^55,56^. In addition, the AMO also showed important influences during the recent warming reacceleration. More detailed investigations should be carried out to study the multiple connections between these factors and their climatic influences.

It is interesting to note that there are differences and similarities in the responses of the above East Asia–Pacific climatic systems to the reacceleration and acceleration of offshore China surface warming. The tropical easterly is enhanced during the warming acceleration period, but it is not strengthened when the warming reacceleration is only located in the ECS region north of 20°N. However, an anomalous cyclonic disturbance occurs in the mid-latitude westerlies over the Chinese continent north of the Yangtze River under warming reacceleration, while it does not appear under warming acceleration. The low-level cyclonic disturbance results in local positive convection anomalies instead of negative convection anomalies during the warming acceleration period. Consequently, SST-induced convective activity controls almost the entire continent on the map (Fig. 4c) and part of the Yellow Sea. On the other hand, the warming reacceleration leads to an interdecadal enhancement of the WPSH, as does the warming acceleration. Hence, a series of interdecadal reactions caused by the interdecadal changes in the WPSH during the warming acceleration period might reappear, such as increased precipitation over the Yangtze River Valley, significant warming in southern China, an increased (decreased) number of tropical cyclones in the region north (south) of 20°N^57^ and weakened monsoons^58^. In addition, the observed oceanic surface warming reacceleration supports the model predictions that the SST is going to warm throughout the twenty-first century because the mean warming rate of the global ocean (0.24 °C/decade) is so close to the rates in tropical storm basins (0.10–0.30 °C/decade), as forecasted by Knutson and Tuleya^59^. Given the surface warming trends, the marine ecosystem would face risks such as frequent outbreaks of harmful algae blooms, which has happened recently^29^.

Since offshore China will continue to warm in the future under global warming, it is worth investigating the response of the East Asia–Pacific climate under the warming reacceleration period more extensively and incisively to understand and forecast East Asia–Pacific climatic changes. More focused discussion of the data gaps and potential impacts, such as the summer monsoon and typhoon responses, of the climatic effects of surface warming reacceleration in offshore China is still required.

## Methods

### Observational data

Several datasets were used in this study for the period 1979–2019. The interdecadal variation in the offshore China SST was analyzed using the HadISST^31^ monthly SST dataset with a horizontal resolution of 1° × 1°. To confirm the results in the present study, the ERSSTv5 and COBE-SST SST datasets were further examined. The ERSSTv5^32^ monthly SST dataset is obtained from NOAA and has a horizontal resolution of 2° × 2°. The COBE-SST^33^ monthly SST dataset is obtained from the Japanese Oceanographic Data Center and has a horizontal resolution of 1° × 1°. The SSTA in the present study is the annual mean of the monthly SST anomalies, which was calculated as the departure of the monthly SST from its long-term mean.

Three merged land–ocean temperature datasets are employed to show global warming based on the GMST, including the HadCRUT^34^, GISTEMP^35^ and NOAAGlobalTemp^36^ datasets.

The ocean currents were obtained from the SODA 3.4.2 dataset^41^ with a 0.5° × 0.5° horizontal resolution and 50 vertical levels, covering the period 1980–2018.

Moreover, the monthly OLR field data from the NOAA^60^ and 850-hPa horizontal winds extracted from the National Centers for Environmental Prediction/National Center for Atmospheric Research (NCEP/NCAR) reanalysis dataset^61^ were used to analyze the climatic response to the interdecadal variation in the offshore China SST.

### Analysis methods

The present study performed an EOF analysis method to analyze the spatial and temporal changes in the offshore China SST. On this basis, we have employed the method in Yao et al.^2^ to identify the different warming stages. The warming acceleration period can be detected by finding the maximum significant tendency during different time interval selections, which has been employed successfully to distinguish distinct global warming periods. The IPO index was calculated as the time coefficient of the EOF2 mode of low-pass-filtered (a 19-point Lanczos filter with a 13-year cutoff period) SSTAs field in the Pacific Ocean^62^, based on the HadISST dataset. The PDO index was calculated as the time coefficient of the EOF1 mode of monthly SSTAs over the North Pacific (after removing the global mean SSTA)^45^. The NPO index was calculated as the time coefficient of the EOF2 mode of the boreal winter SLP anomalies in the North Pacific region^63^. The AMO, ENSO (Niño 3.4), AO and IOD indices are derived from https://stateoftheocean.osmc.noaa.gov/sur/. All aforementioned annual indices are standardized by dividing their standard deviations during 1979–2019 in the present study. In addition, the influence of the interdecadal SST variation on the atmospheric circulation and convection in offshore China was further assessed by the linear regression analysis. In addition, Student’s *t* test was used to examine the significance of the linear trend and regression coefficient. The confidence interval was set to 90% in this study.

## Supplementary information

Supplementary Information 1.
