# Supplementary material for: Surface warming reacceleration in offshore China and its interdecadal effects on the East Asia–Pacific climate
Source: Sci Rep. 2020 Sep 9;10:14811. doi: 10.1038/s41598-020-71862-6 (PMC7481234; doi:10.1038/s41598-020-71862-6)
Supplement: Supplementary file 1 — Supplementary Information 1. [file 41598_2020_71862_MOESM1_ESM.pdf]

Surface warming reacceleration in offshore China and its interdecadal effects on the East Asia-Pacific climate

Yulian Tang[1, 2], Jingliang Huangfu[1, 2*], Ronghui Huang[1], and Wen Chen[1, 2]

[1]Center for Monsoon System Research, Institute of Atmospheric Physics, Chinese Academy of Sciences, Beijing 100190, China

[2]University of Chinese Academy of Sciences, Beijing 100049, China

[*]hfjl@mail.iap.ac.cn

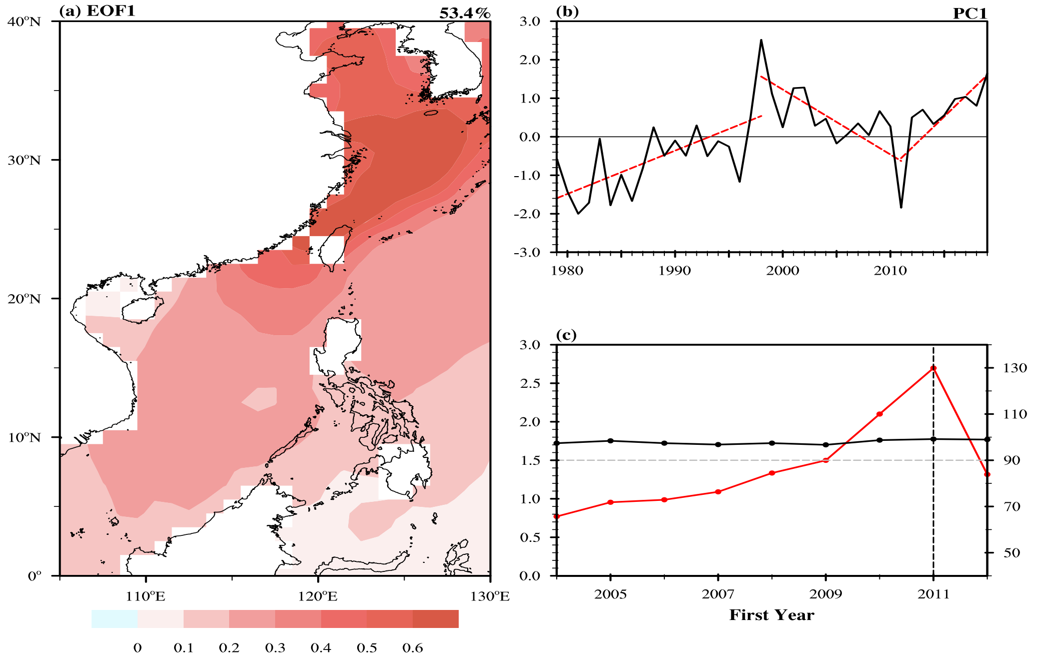

Supplementary Figure 1 (a) The leading EOF1 for the boreal spring (MAM) SST anomalies in offshore China during 1979-2019 and (b) its PC1 based on the HadISST dataset. The red line in (c) represents the linear tendency of PC1 with different time interval selections, and each year on the X-axis denotes a period between itself and 2019. The black line in (c) represents the relevant confidence level (unit: %) according to a one-sided Student's t-test. The map in figure (a) is generated using the NCAR Command Language (Version 6.6.2) [Software]. (2019). Boulder, Colorado: UCAR/NCAR/CISL/TDD. http://dx.doi.org/10.5065/D6WD3XH5.

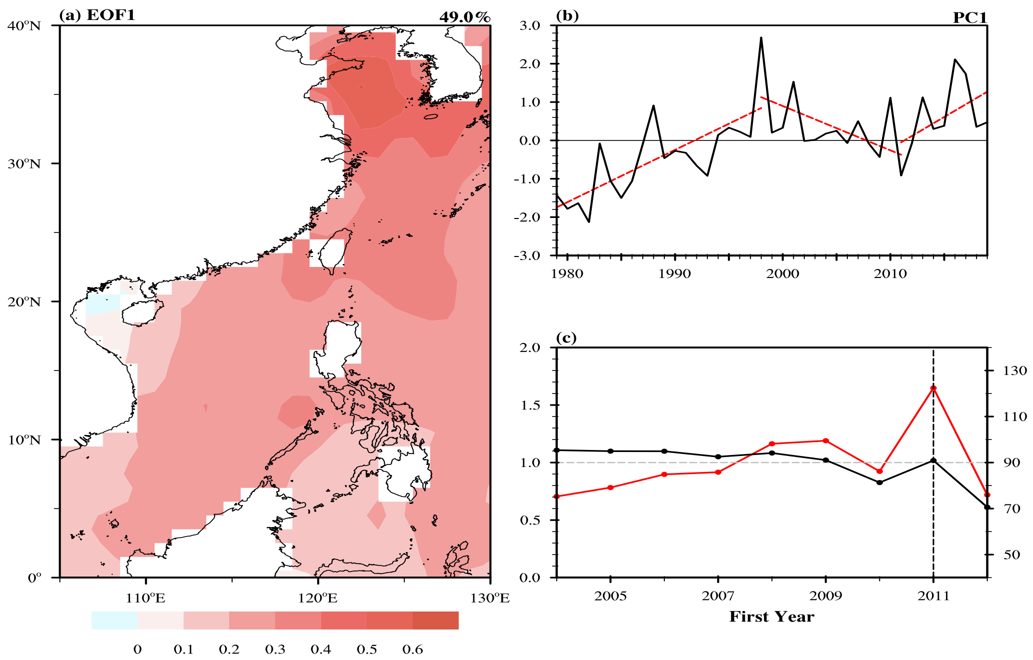

Supplementary Figure 2 The same as Supplementary Figure 1, but for the boreal summer (JJA). The map in figure (a) is generated using the NCAR Command Language (Version 6.6.2) [Software]. (2019). Boulder, Colorado: UCAR/NCAR/CISL/TDD. http://dx.doi.org/10.5065/D6WD3XH5.

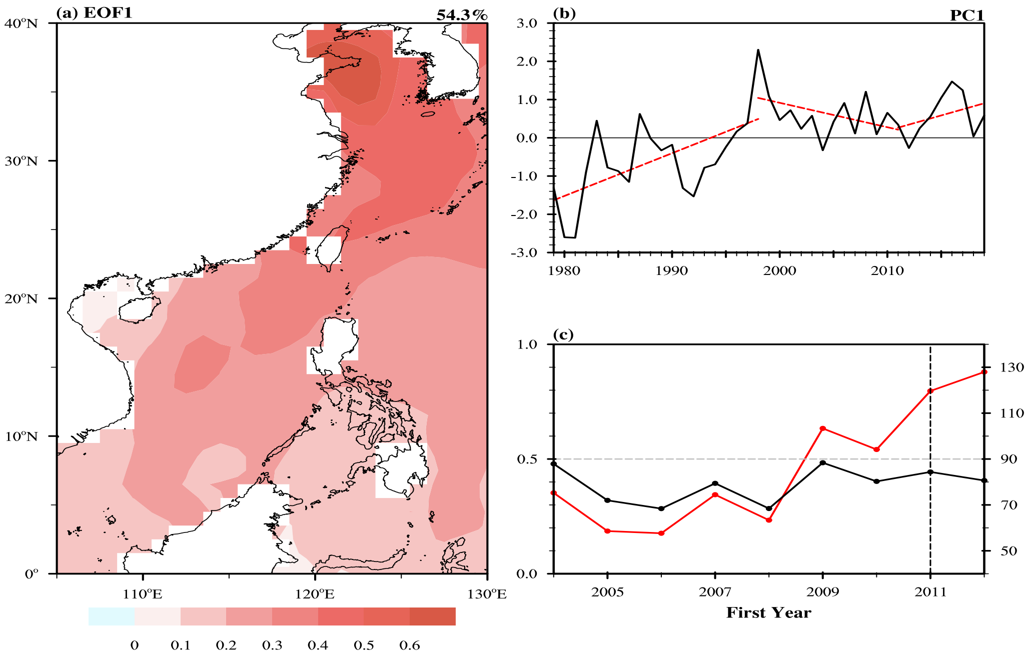

Supplementary Figure 3 The same as Supplementary Figure 1, but for the boreal autumn (SON). The map in figure (a) is generated using the NCAR Command Language (Version 6.6.2) [Software]. (2019). Boulder, Colorado: UCAR/NCAR/CISL/TDD. http://dx.doi.org/10.5065/D6WD3XH5.

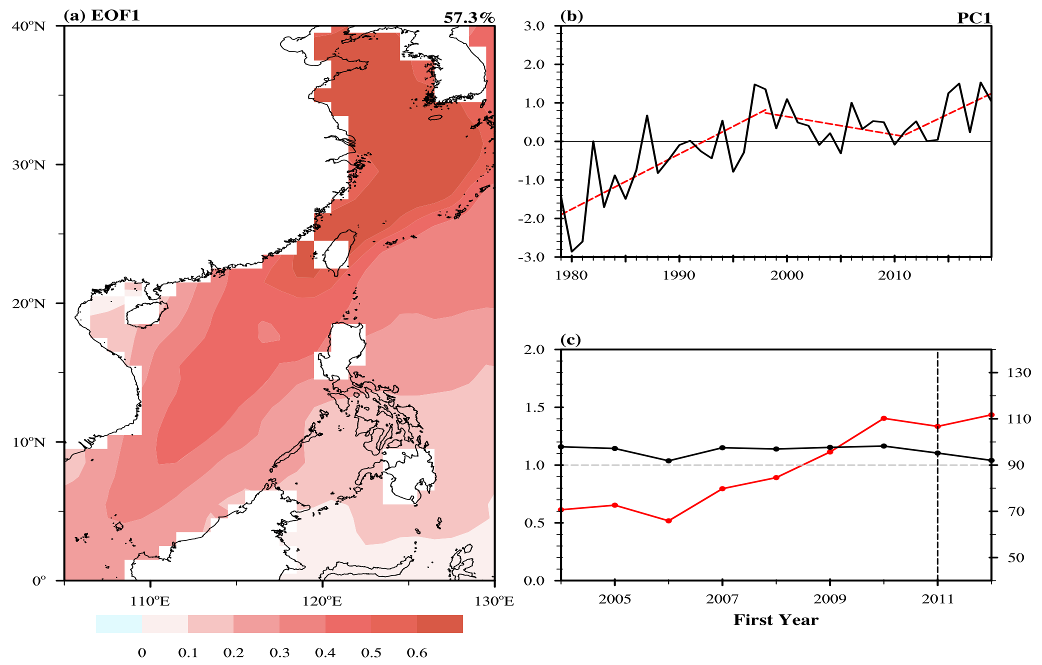

Supplementary Figure 4 The same as Supplementary Figure 1, but for the boreal winter (DFJ). The map in figure (a) is generated using the NCAR Command Language (Version 6.6.2) [Software]. (2019). Boulder, Colorado: UCAR/NCAR/CISL/TDD. http://dx.doi.org/10.5065/D6WD3XH5.

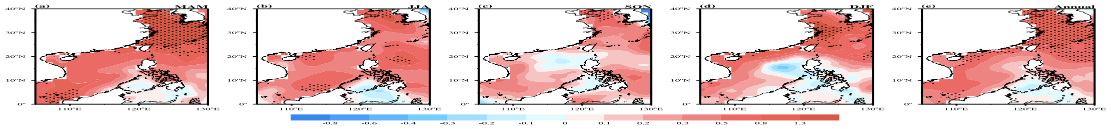

Supplementary Figure 5 Linear trends of the boreal (a) spring (MAM), (b) summer (JJA), (c) autumn (SON), (d) winter (DJF) and (e) annual mean SSTAs (unit: °C/decade) in offshore China during the warming reacceleration period (2011-2019) based on the HadISST dataset. Stippling denotes 10% significance according to a two-sided Student's t-test. The maps in the figure are generated using the NCAR Command Language (Version 6.6.2) [Software]. (2019). Boulder, Colorado: UCAR/NCAR/CISL/TDD. http://dx.doi.org/10.5065/D6WD3XH5.

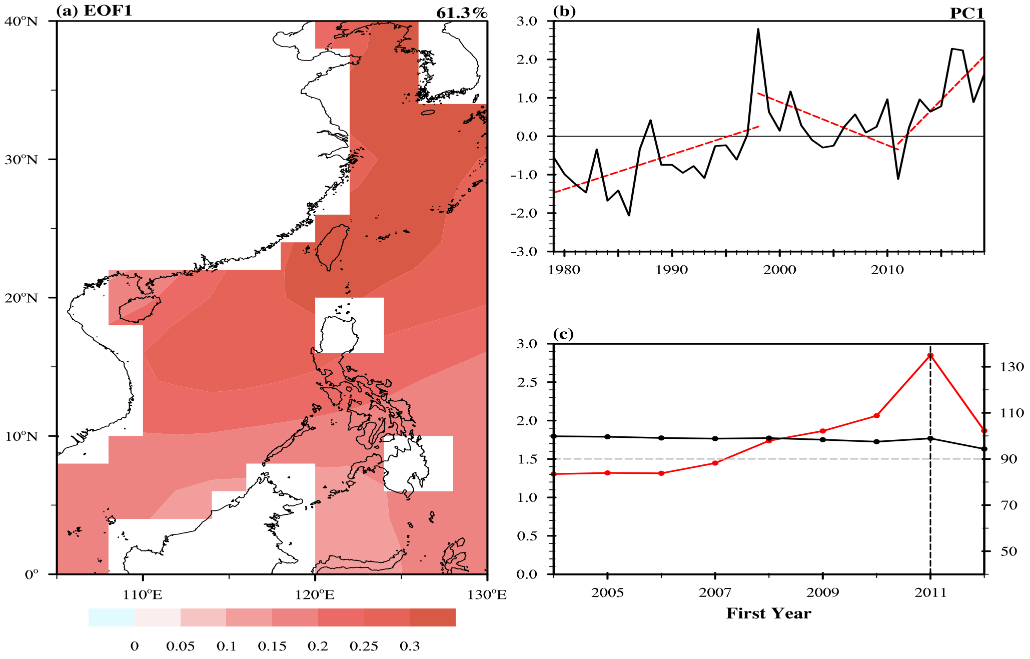

Supplementary Figure 6 The same as Supplementary Figure 1 but for the annual mean SST anomalies in offshore China based on the ERSSTv5 dataset. The map in figure (a) is generated using the NCAR Command Language (Version 6.6.2) [Software]. (2019). Boulder, Colorado: UCAR/NCAR/CISL/TDD. http://dx.doi.org/10.5065/D6WD3XH5.

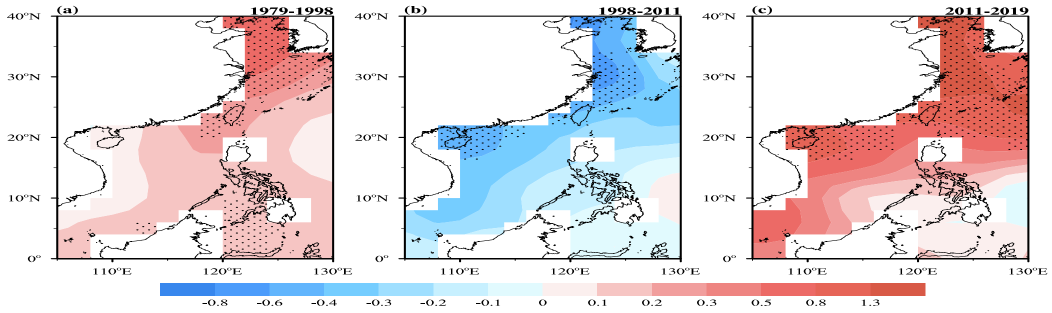

Supplementary Figure 7 Spatial patterns of the linear trends of the annual mean SST anomalies in offshore China during (a) the warming acceleration period (1979-1998), (b) the warming slowdown period (1998-2011) and (c) the warming reacceleration period (2011-2019) based on the ERSSTv5 dataset. Stippling denotes 10% significance according to a two-sided Student's t-test. The maps in the figure are generated using the NCAR Command Language (Version 6.6.2) [Software]. (2019). Boulder, Colorado: UCAR/NCAR/CISL/TDD. http://dx.doi.org/10.5065/D6WD3XH5.

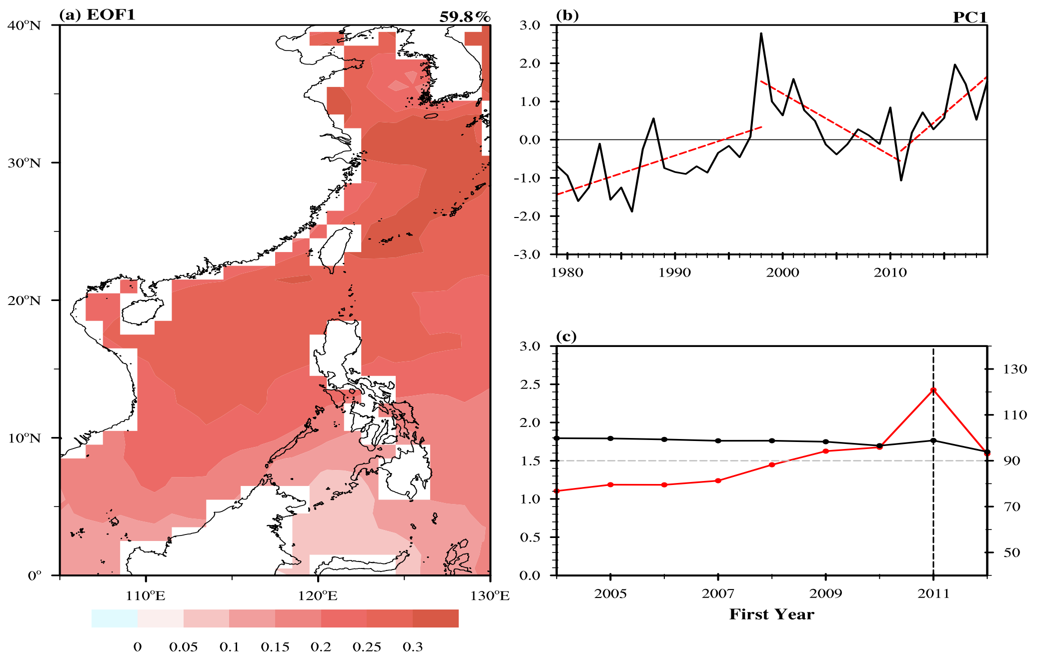

Supplementary Figure 8 The same as Supplementary Figure 1 but for the annual mean SST anomalies in offshore China based on the COBE-SST dataset. The map in figure (a) is generated using the NCAR Command Language (Version 6.6.2) [Software]. (2019). Boulder, Colorado: UCAR/NCAR/CISL/TDD. http://dx.doi.org/10.5065/D6WD3XH5.

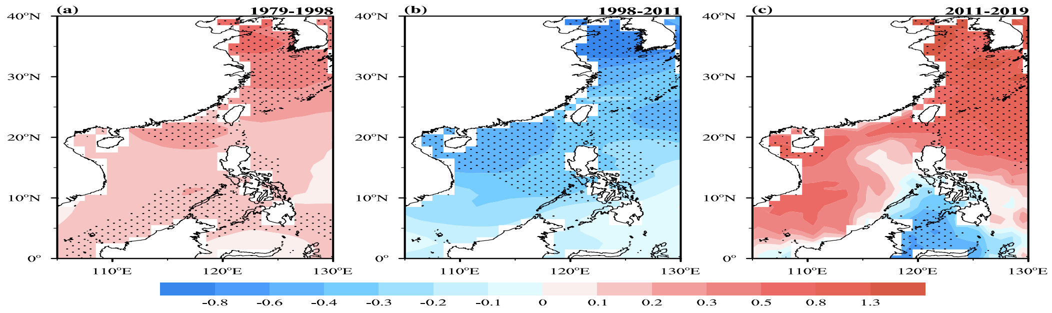

Supplementary Figure 9 The same as Supplementary Figure 7 but based on the COBE-SST dataset. The maps in the figure are generated using the NCAR Command Language (Version 6.6.2) [Software]. (2019). Boulder, Colorado: UCAR/NCAR/CISL/TDD. http://dx.doi.org/10.5065/D6WD3XH5.

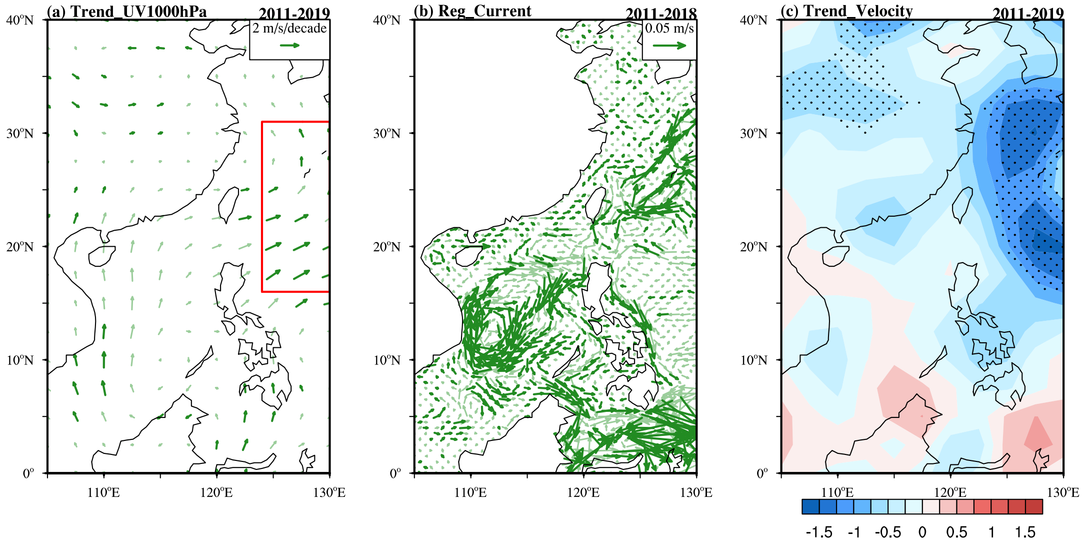

Supplementary Figure 10 (a) The linear trend of the 1000-hPa wind field (vector; unit: m/s/decade) during 2011-2019. (b) The regression of the ocean circulation upper 30 m from the SODA 3.4.2 dataset onto the 1000-hPa wind anomalies over the red box region (16°N-31°N, 124°E-130°E) during 2011-2018. (c) The linear trend of the velocity of 1000-hPa winds over offshore China during 2011-2019. The dark green vectors and black dots denote 10% significance according to a two-sided Student's t-test.

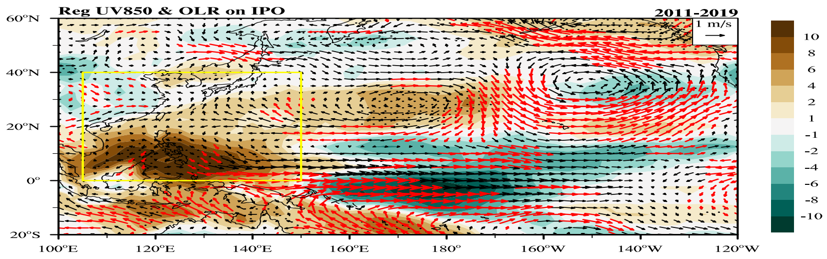

Supplementary Figure 11 Spatial distribution of the regression of the 850-hPa wind field (vector; unit: m/s) and OLR field (shaded; unit: W/m^2) on the IPO index during the warming reacceleration period (2011-2019). The dots and red vectors denote regression significance at the 90% confidence level. The yellow box indicates the coverage of the map in Fig.4 in the Manuscript (0°-40°N, 105°E-150°E). The map in the figure is generated using the NCAR Command Language (Version 6.6.2) [Software]. (2019). Boulder, Colorado: UCAR/NCAR/CISL/TDD. http://dx.doi.org/10.5065/D6WD3XH5.
